# Are the results of questionnaires measuring non-cognitive characteristics during the selection procedure for medical school application biased by social desirability?

**DOI:** 10.3205/zma001074

**Published:** 2016-11-15

**Authors:** Katrin U. Obst, Linda Brüheim, Jürgen Westermann, Alexander Katalinic, Thomas Kötter

**Affiliations:** 1 University of Lübeck, Institute of Social Medicine and Epidemiology, Lübeck Germany; 2 University of Lübeck, Department of Quality Management and Organizational Development, Lübeck Germany; 3 University of Lübeck, Institute of Anatomy, Lübeck Germany

**Keywords:** Medical Education, College Admission Test, Psychometrics, Personality Inventory, Social Desirability

## Abstract

**Introduction: **A stronger consideration of non-cognitive characteristics in Medical School application procedures is desirable. Psychometric tests could be used as an economic supplement to face-to-face interviews which are frequently conducted during university internal procedures for Medical School applications (AdH, Auswahlverfahren der Hochschulen). This study investigates whether the results of psychometric questionnaires measuring non-cognitive characteristics such as personality traits, empathy, and resilience towards stress are vulnerable to distortions of social desirability when used in the context of selection procedures at Medical Schools.

**Methods: **This study took place during the AdH of Lübeck University in August 2015. The following questionnaires have been included: NEO-FFI, SPF, and AVEM. In a 2x1 between-subject experiment we compared the answers from an alleged application condition and a control condition. In the alleged application condition we told applicants that these questionnaires were part of the application procedure. In the control condition applicants were informed about the study prior to completing the questionnaires.

**Results: **All included questionnaires showed differences which can be regarded as social-desirability effects. These differences did not affect the entire scales but, rather, single subscales.

**Conclusion: **These results challenge the informative value of these questionnaires when used for Medical School application procedures. Future studies may investigate the extent to which the differences influence the actual selection of applicants and what implications can be drawn from them for the use of psychometric questionnaires as part of study-place allocation procedures at Medical Schools.

## Zusammenfassung

**Zielsetzung: **Eine stärkere Berücksichtigung nicht-kognitiver Eigenschaften in der Studienplatzvergabe im Fach Medizin wäre prinzipiell wünschenswert. Der Einsatz psychometrischer Fragebögen könnte eine ökonomische Ergänzung zum Auswahlgespräch, das vielerorts Teil des Auswahlverfahrens der Hochschulen (AdH) ist, darstellen. Diese Studie untersucht, inwiefern Ergebnisse psychometrischer Fragebögen zur Erfassung nicht-kognitiver Eigenschaften wie Persönlichkeit, Empathie und Stress-Resilienz anfällig für Verzerrungen durch sozial erwünschtes Antwortverhalten sind, wenn Sie im Kontext der Studienplatzvergabe im Fach Medizin eingesetzt werden.

**Methodik: **Die Studie fand im Rahmen des AdH der Universität zu Lübeck im August 2015 statt. Untersucht wurden folgende Fragebögen: NEO-FFI, SPF und AVEM. In einem experimentellen 2x1-Zwischensubjekt-Design wurde das Antwortverhalten zwischen einer vermeintlichen Auswahlbedingung und einer Kontrollbedingung verglichen. In der Auswahlbedingung wurde den Bewerberinnen und Bewerbern suggeriert, dass die Fragebögen Teil des Auswahlprozesses seien. In der Kontrollbedingung wurde im Vornherein mitgeteilt, dass die Fragebögen Teil einer wissenschaftlichen Begleitstudie sind.

**Ergebnisse: **Für alle untersuchten Fragebögen weisen die Ergebnisse Unterschiede auf, die auf soziale Erwünschtheit hindeuten. Dabei sind nicht die gesamten Fragebögen betroffen, sondern einzelne Subskalen.

**Schlussfolgerung:** Die Aussagekraft der untersuchten Fragebögen für den Einsatz in der Auswahlsituation muss in Frage gestellt werden. Zukünftige Studien müssen untersuchen, inwiefern sich die hier gefundenen Unterschiede tatsächlich auf den Auswahlprozess auswirken und welche Implikationen sich daraus für deren Einsatz bei der Studienplatzvergabe im Fach Medizin ergeben.

## 1. Introduction

For many years, there have been up to five graduates applying for each place available at Medical School [1]. So far, Abitur (final exam) grades were considered the main criterion for study-place allocation. Taking additional selection criteria into account, such as clinical or non-cognitive characteristics, universities allocate up to 60% of their study places via university internal procedures for Medical School applications (AdH, Auswahlverfahren der Hochschulen). However, even in the AdH, the Abitur grade is the most important criterion [2]. This state of affairs has been criticized by many stakeholders and a reform of these application procedures has been demanded [3], [4], [http://www.aerzteblatt.de/nachrichten/sw/Studienplatzvergabe?nid=63689].

Although the Abitur grade predicts study grades as well as whether the standard period of study can be maintained by individuals [5], [6], [7], using this as the main criterion is problematic because it does not tell us anything about the characteristics that define a “good physician”, including clinical skills, empathy, resilience to stress, and certain personality traits [8]. These characteristics are desirable for faculty and society. In fact, studies show that these characteristics predict study success on the one hand and, on the other hand, who may become a “good physician” [9], [10]. Voltmer et al. show that work-related behavior and experience patterns have an impact on the health of medical students and young physicians [11], [12], [13]. Health is a basic prerequisite for the professional and empathetic treatment of patients [14], [15]. Physicians’ empathy appears to enhance patient satisfaction and also influences the correctness of diagnoses and treatment success in a positive way [16]. Furthermore, certain personality traits, such as* openness to experience* or *agreeableness*, are associated with the ability to empathize [17] and linked directly to patient satisfaction [18] and study success [19].

To identify non-cognitive characteristics at some German universities, including Lübeck University, face-to-face interviews are an integral part of the AdH (e.g., [20], [21], [22]). These guideline-based interviews are conducted by a commission. However, they are resource-consuming and doubtful in their validity [23]. For these reasons, the use of supplementary validated questionnaires is advised [24]. Experience with the use of questionnaires to measure non-cognitive characteristics during the AdH is rare. These questionnaires are, however, well validated in the contexts of research studies [25], [26], [27]. It remains open as to whether such questionnaires are influenced by social desirability when answered in the context of Medical School application procedures [28], [29]. In this case answers to the questionnaires would have little meaning and would not be of much help in deciding to whom a study place should be given. Therefore, before AdH procedures are supplemented by questionnaires to measure non-cognitive characteristics, these should be tested for distortions of social desirability. Low distortions of social desirability are a prerequisite for using such instruments during AdH procedures. 

On the basis of the aforementioned studies discussing which characteristics predict who may become a “good physician”, this study concentrates on measures of empathy, personality traits, and work-related behavior and experience patterns. The main question of this study is: Are questionnaires which measure these non-cognitive characteristics prone to social desirability distortion?

We expect that applicants of the AdH show enhanced tendencies to present themselves in a socially-desirable way and in accordance with what they think characterizes a “good physician”.

## 2. Methods

### 2.1 Participants

This study investigated applicants for Medical School at Lübeck University who participated in the face-to-face interviews which took place as part of the AdH 2015. Overall, 240 applicants were invited to the interviews in 2015; 228 accepted the invitation. 226 of these were included in our study (see table 1 (Tab. 1)) and two did not participate. 

### 2.2 Employed instruments

The following questionnaires have been tested for the applicability in the AdH.

#### SPF

The SPF [25] is a German translation and revision of the Davis Interpersonal Reactivity Index [30]. Empathy is measured through four facets:

1. Perspective taking (ability to adopt another’s point of view),
2. fantasy (ability to empathize with fictional characters),
3. empathic concern (ability for other-oriented emotions, such as pity), and
4. personal distress (self-oriented emotions such as uneasiness, which may occur in close or problematic interpersonal interactions).

Being a self-oriented emotion, personal distress is not considered in the empathy score.

For the medical context, the Jefferson Scale of Physician Empathy (JSPE [31]) might be more common. However, its wording makes it easy to guess what it measures, which makes it particularly vulnerable to social-desirability effects. This is why we decided to use a less obvious measurement of empathy, even though this measure is not customized to the medical context. 

#### NEO-FFI

The NEO-FFI by Costa & McCrae (German translation by Borkenau & Ostendorf [26]) is a personality questionnaire which measures personality according to the Big Five Model. Personality is described by five independent factors: neuroticism, extraversion, openness to experience, conscientiousness, and agreeableness.

*Neuroticism* comprises characteristics such as nervousness and uncertainty, but also inappropriate reactions to stress. *Extraversion* describes, among other characteristics, the extent of the sociability and optimism a person has. *Openness to experience* measures to what extent new experiences or changes are welcomed as well as creativity and a thirst for knowledge. *Agreeableness* includes social emotion and characteristics such as altruism, faith, cooperation, and softness. *Conscientiousness* comprises characteristics such as orderliness, reliability, and discipline.

Individuals are asked to indicate their agreement or disagreement with 60 items on a five-point Likert Scale. Means have been calculated for the items of each factor.

#### AVEM

The AVEM [27] is a multidimensional personality-diagnostics procedure which measures behavior and experiences with regard to job-related stress and its influences on health. Self-reports are gathered on a five-point Likert Scale according to the following 11 dimensions:

1. subjective significance of work;
2. career ambition;
3. tendency to exert;
4. striving for perfection;
5. emotional distancing;
6. resignation tendencies;
7. offensive coping with problems;
8. balance and mental stability;
9. satisfaction with work;
10. satisfaction with life;
11. experience of social support.

Based on the interplay of these 11 dimensions, four profiles, or patterns of behavior and experiences can be derived by means of cluster analysis:

- Pattern G (“health”): high but not excessive work commitment, high resilience and positive emotion, including a high satisfaction with life and experience of social support.
- Pattern S (“unambitious”): reduced work commitment combined with a high ability to distance from work and a high satisfaction with life.
- Risk pattern A (”overexertion”): high work commitment but low resilience, satisfaction with life is impaired.
- Risk pattern B (“burnout”): low work commitment, especially low significance of work and low career ambition, combined with low ability to distance from work, high tendencies to resign, low satisfaction with life.

For this study we used the short version of AVEM with 44 items. Wordings were adapted to the students’ situation.

All data collected in this study constitute self-reports and not objectively-measured characteristics.

### 2.3 Study design

Applicants were randomly administered to one of two experimental conditions (one factorial, experimental design). At each stage, 12 applicants had their application interview simultaneously. We assigned these 12 simultaneously-interviewed applicants to one of the two experimental conditions in an alternating fashion. They were then asked to complete a questionnaire at the computer. The allocation of interview time slots for each applicant was realized by means of computer-generated random numbers and this was conducted by the interview organization team. 

Applicants in the first group (*alleged application condition*) were persuaded by instruction that their answers to the questionnaire would be considered in their application for Medical School in the event that the interview result was equivocal (excerpt of instruction: In the case of equivocal judgments of the commission, the results of this questionnaire will help us to allocate the study places among the invited applicants in a fair way). Study information was given immediately after completion of the questionnaire.

Applicants in the control condition (*study condition*) were informed about the study and its aim prior to completing the questionnaire (excerpt of instruction: We would like to invite you to complete a set of questions which will help us to optimize study-place allocation procedures in the future).

Participants of this *study condition* had not been informed about the aim to test the questionnaires for social-desirability influences. However, it was explicitly pointed out that the answers to the questionnaire had no influence on interview results or their application to Medical School. 

This experimental design allows comparison of the questionnaire answers under a condition of personnel selection, which decides the future life of the applicants with regard to the answers of the same questionnaires used in the common research condition. As such, we wanted to test whether, and to what extent, answers to our selected questionnaires might be changed by a personnel selection situation and, thus, whether our questionnaires were prone to social-desirability effects [28], [29].

By means of an additional scale for social desirability (SES-17, [32]), we tested whether the *alleged application condition* enhanced social-desirability tendency in general compared to the *study condition*. This scale functions as a control for the experimental manipulation.

At the end of the questionnaire, applicants had the opportunity to make comments about the study. These comments are included in the data analysis.

### 2.4 Analysis

Questionnaire data were imported into and analyzed with the statistic software IBM SPSS Statistics (version 22).

All group comparisons were statistically controlled for age, gender, and Abitur grade. To control for alpha cumulation due to multiple testing, data were Bonferroni-corrected for each questionnaire.

### 2.5 Ethics

To maintain the deception, we could not inform applicants in the *alleged application condition* about the voluntariness of their participation in this study in advance. Immediately after completion of the questionnaire, however, applicants were informed, orally and in written form, about the study and its aims. Additionally, we sought their written consent. In the case of no written consent we withdrew the data set. However, all participants who completed the questionnaire also gave their written consent to the study. The procedure was discussed and authorized by the Ethical Committee of the University of Lübeck (file reference: 15-072).

## 3. Results

Firstly, we tested whether applicants in the *alleged application condition* showed higher scores for the social-desirability scale (SES-17) than applicants in the *study condition*. We could not show a statistically significant difference between the two experimental conditions (*study condition*: *M*=13.11, *SD*=2.78 vs. *alleged application condition*: *M*=13.42, *SD*=2.42, *F[1,220]*=0.53, n.s.).

**Figure 1:**
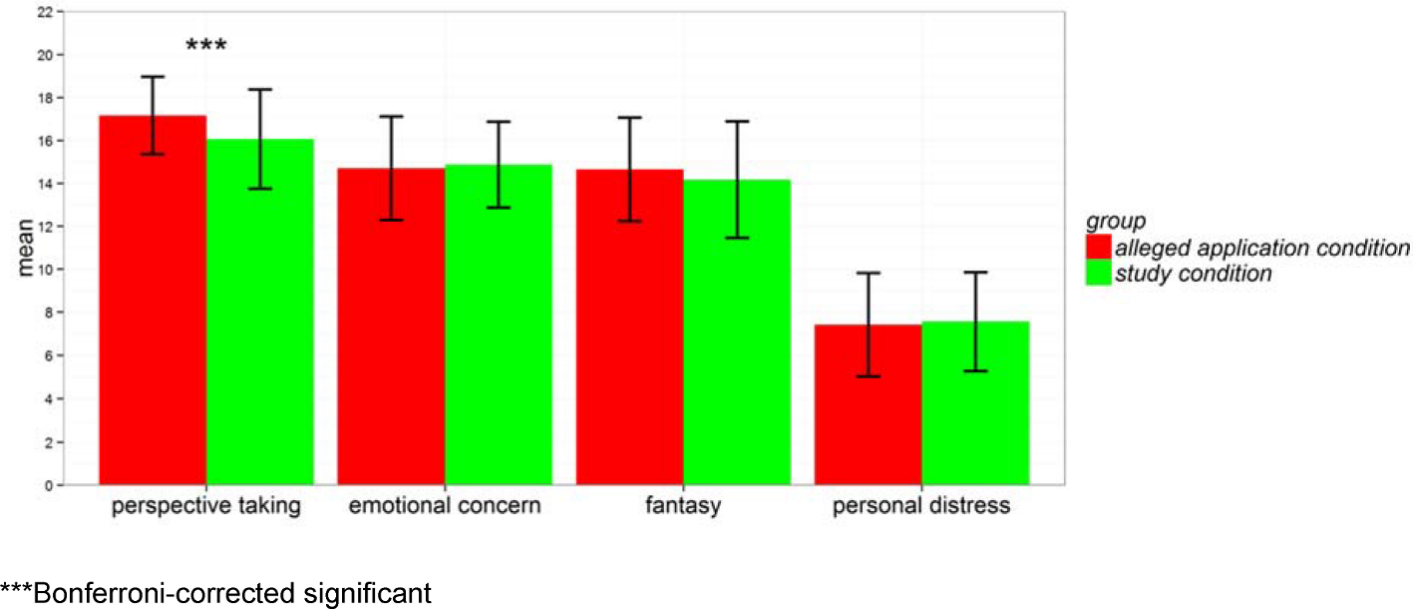
Mean comparison of the empathy scale SPF for the two experimental conditions: *alleged application condition *vs.* study condition*

**Figure 2:**
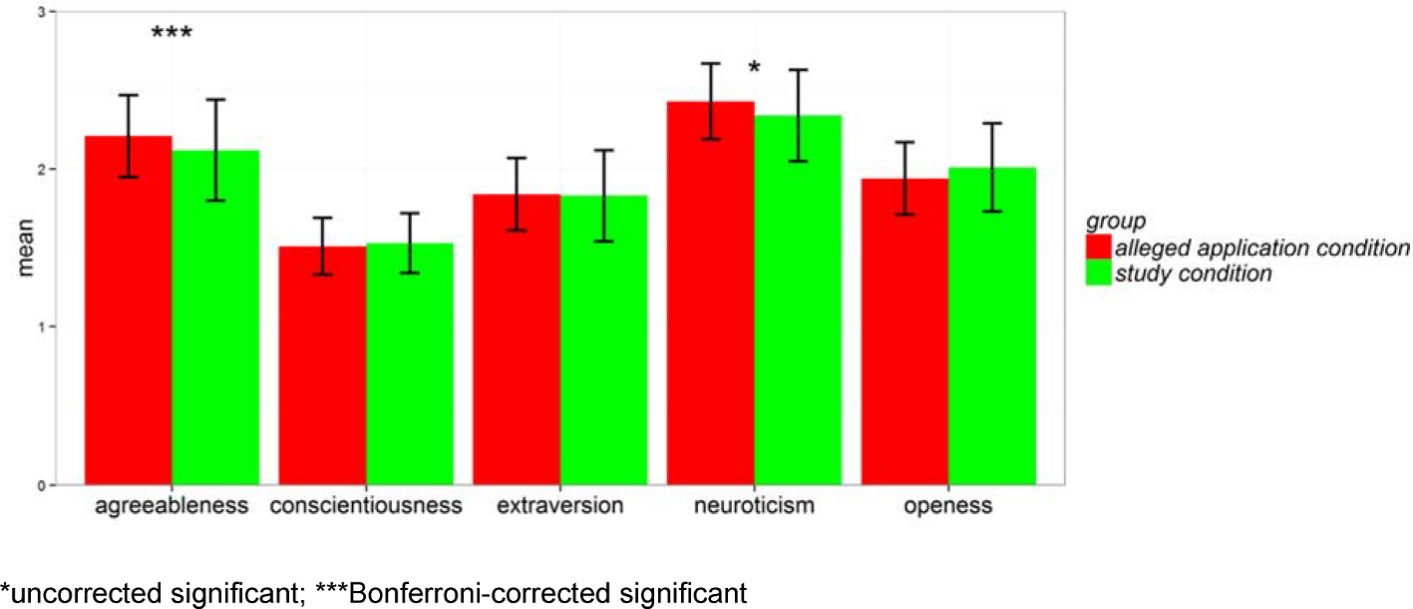
Mean comparison of the NEO-FFI for the two experimental conditions: *alleged* *application condition* vs. *study condition*

**Figure 3:**
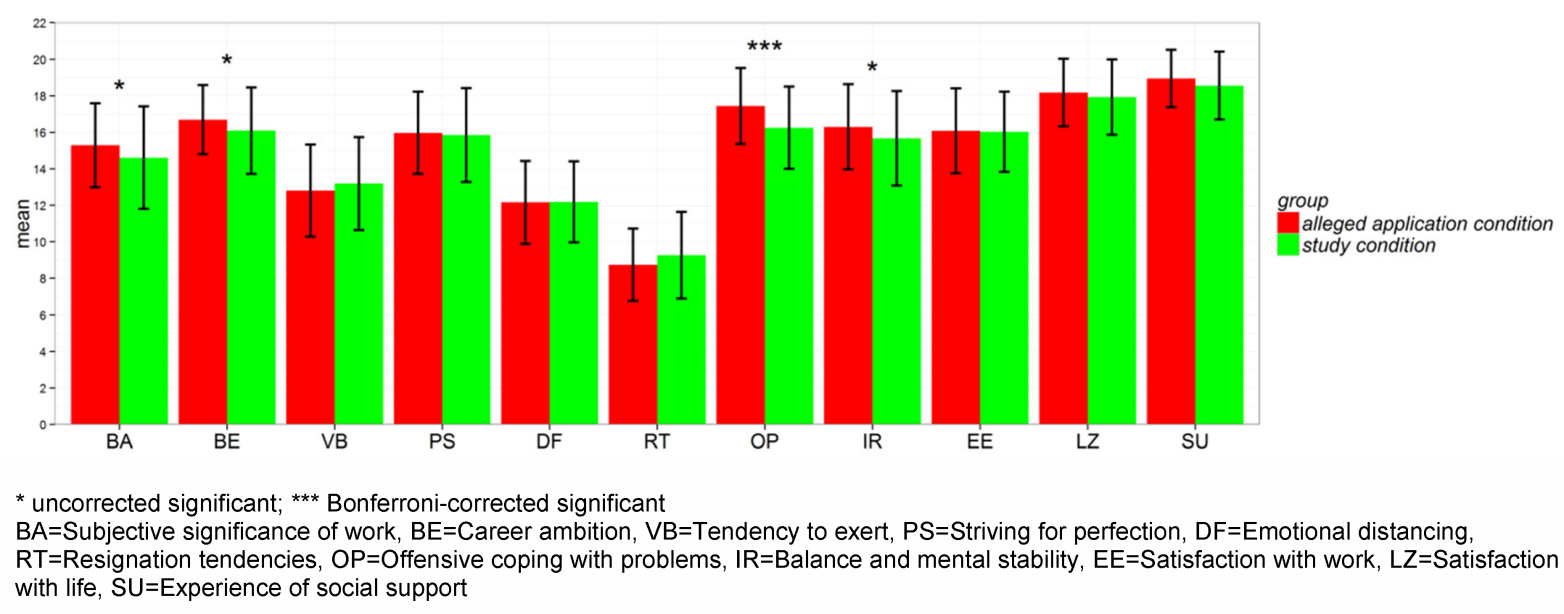
Mean comparison of the AVEM for the two experimental conditions: *alleged application condition* vs. *study condition*

Nevertheless, there were differences between the two experimental conditions for all questionnaires that might be regarded as social-desirability effects (see Figure 1 (Fig. 1), Figure 2 (Fig. 2), Figure 3 (Fig. 3) and Table 2 (Tab. 2)).

Accordingly, the empathy score differs significantly. Applicants of the *alleged application condition* seem to be significantly more empathic than applicants of the *study condition*. Examining the facets of the SPF more closely it becomes obvious that this difference relies mostly on a significant difference in the *perspective-taking* facet (see Figure 1 (Fig. 1)).

Moreover, applicants of the *alleged application condition* show higher scores for the factors of neuroticism and agreeableness of the NEO-FFI compared to the *study condition*. The difference in the factor of agreeableness, however, did not withstand Bonferroni correction (see Figure 2 (Fig. 2)).

The 11 dimensions of the AVEM revealed the following results (see Figure 3 (Fig. 3)): applicants of the *alleged application condition* showed higher scores for *offensive coping with problems*. There were also higher scores for *subjective significance of work, career ambition*, and *balance and mental stability*. However, the last three dimensions reached significance only uncorrected. Remarkably, these differences in the AVEM-dimensions’ results showed a three-times higher probability of reaching the AVEM pattern G for applicants in the *alleged application condition* compared to applicants in the *study condition* (*OR*=3.15, *WALD[1]*=5.35, *p*=.03).

Since the other AVEM patterns were relatively rare (pattern S: n=4; risk-pattern A: n=19; risk-pattern B: n=2), we did not analyze them. 

Taken together, we can show differences between the experimental conditions for each scale. These differences have small-to-medium effect sizes (see Table 2 (Tab. 2)).

Expectations about distortion effects are also evident in some of the comments applicants made during the evaluation of the interviews at the end of the day: in total, 20 comments referred to the study. From these, eight comments referred to general aspects, such as appreciation of such studies on the one hand or doubts about their informative values on the other. Four applicants from the *alleged application condition* did not believe that the questionnaire was part of the Medical School Application procedure. Six comments referred to aspects of social desirability. In fact, applicants in both experimental conditions pointed out that questionnaires might be distorted by social desirability when used in the context of study-place allocation (see Table 3 (Tab. 3)). Even though these six comments were not representative, they correspond neatly with the direction of differences that we reported in all questionnaires for our two experimental conditions.

## 4. Discussion

This study tested whether selected questionnaires for measuring personality traits, including empathy and coping with stress, are vulnerable to social-desirability effects when used in the framework of Medical School applications.

Our results show that the scores of all selected questionnaires are indeed distorted when used in the context of Medical School applications. Even after the Bonferroni correction for multiple testing, there were statistically-significant differences in the subscales of all implemented questionnaires. These differences had small-to-medium effect sizes [33]. Moreover, the AVEM differences appear to cumulate, which resulted in a three-times higher probability of reaching the AVEM pattern G for applicants in the *alleged application condition* compared to the *study condition*. This difference is remarkable in and of itself.

The use of personality questionnaires in the framework of personnel selection is controversially discussed in the fields of Personality and Organizational Psychology. Studies in which participants are asked explicitly to present themselves in the best possible fashion (fake good paradigms) show expectation- and stereotype- conforming behavior [34], [35], [36], [37], [38]. According to these studies, personality tests do indeed seem prone to social-desirability effects. However, other studies show only few effects of social desirability on personality tests [39], [40], [41]. Especially when participants are put in seemingly real personnel-selection procedures, the effects of social desirability seem to be rather small [40]. This might explain the small effect sizes reported in this study.

Being a paradigm that does not involve faking goodness may also explain why applicants seem to represent themselves as more neurotic in the *alleged application condition* compared to the *study condition*. Applicants tried to represent themselves in accordance with what a “good” student and a “good” physician might be. This is why applicants probably tried to avoid representing themselves as too self-confident, especially considering the fact that self-reflection is part of the picture of an ideal physician [42]. 

Regarding empathy questionnaires or the AVEM, the authors are not aware of comparable studies investigating social desirability.

Since the number of cases (226) is rather small to reliably detect differences in this range of effect sizes, this study may rather underestimate the influence of social desirability on the selected questionnaires [43]. Thus, although the reported differences might be rather occasional and small at first glance, based on our data we cannot assume that these questionnaires measure personality traits, empathy and stress resilience in a valid way.

However, there is one crucial limitation. Although we can show differences between the two experimental conditions for all selected questionnaires we cannot show a significant difference between the experimental conditions on the social desirability scale. This means that the distortions we could show for the other questionnaires might not be due to social desirability at all. Regarding our data, we could not rule out this limitation. The SES-17 [32] scale used in this study measures social desirability by very global everyday situations that have little to do with Medical School applications or personnel selection in general. Social desirability, on the other hand, describes a behavioral tendency to conform as best as possible within situation-specific norms and expectations [29]. Thus, it is possible that the Medical School application procedure triggers social desirability in a more context-specific way for which the SES-17 scale might not be sensitive enough.

Moreover, a stronger wording of the instruction text in the *alleged application condition* (see Methods) might have enhanced differences between the experimental conditions. Regarding their direction, the differences do indicate that applicants of the *alleged application condition* strived to represent themselves in terms of an ideal student and ideal physician. This interpretation is also supported by the open-text comments of the evaluation of the Medical School application interviews. Taken together, this indicates that the SES-17 scale was probably not an appropriate control scale.

Overall, the results reported in this study show that psychometric questionnaires should be used with caution for Medical School application procedures, although their use might be highly desirable. We recommend further evaluating and probably re-editing psychometric questionnaires to reduce their vulnerability for social-desirability effects. Whether the differences shown here affect the actual selection procedure remains an open question. For example, it is conceivable that applicants with high tendencies of social desirability might reach higher ranks and thus have a higher probability in gaining the desired study place. Furthermore, it remains open as to what implications this could have regarding the matter of how to select those applicants who have the highest probability of becoming “good” physicians [44]. The results reported here make clear that research in this field is desperately needed regarding when psychometric questionnaires should be used in Medical School application procedures. 

## 5. Conclusions

- Using psychometric questionnaires for Medical School application procedures is not problem-free: questionnaires tested in this study show distortions of social desirability. However, if answers are influenced by a desire to represent themselves in terms of the ideal student or physician, the answers lose their predictive value and, thus, are of no use for study-place allocation.
- Future research is required to evaluate the actual impact of those social-desirability effects and to reveal alternative procedures.

## Acknowledgements

We thank Jessica Lückert, Sophia Marie Saftien, Karl Böse, Karen Sievers, and Josefin Wagner for their support in the preparation and implementation of this study.

## Competing interests

The authors declare, that they have no competing interests.

As part of her job role at Lübeck University, Linda Brüheim was involved in the analyses of the Medical School application procedures (AdH). 

The study was funded by Lübeck University, Medical School Lübeck and the Institute of Social Medicine and Epidemiology, Lübeck University.

## Figures and Tables

**Table 1:**
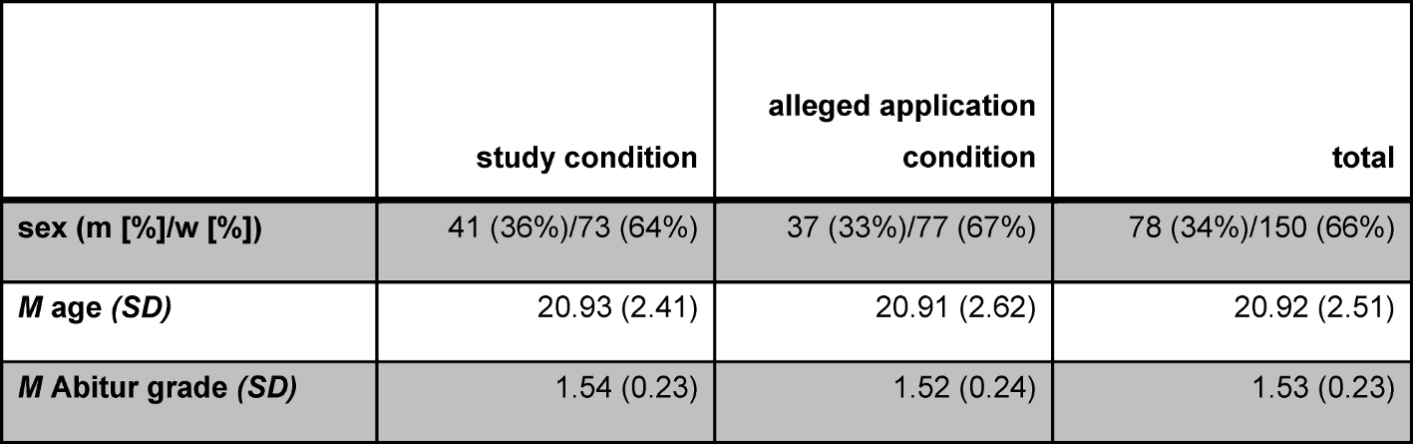
Socio-demographic data and Abitur grade

**Table 2:**
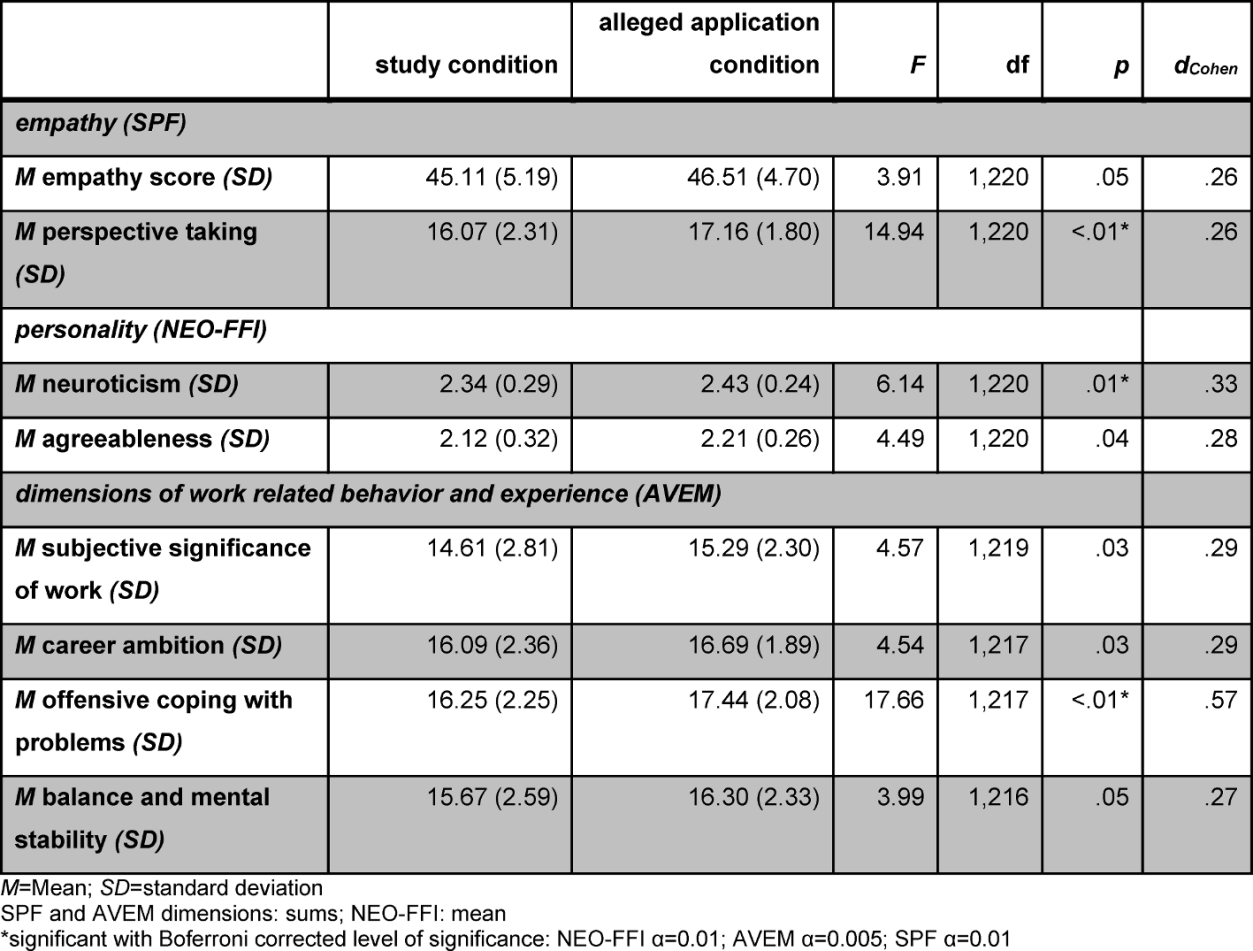
Statistical characteristics for significant differences in empathy, personality traits, and work-related behavior and experiences

**Table 3:**
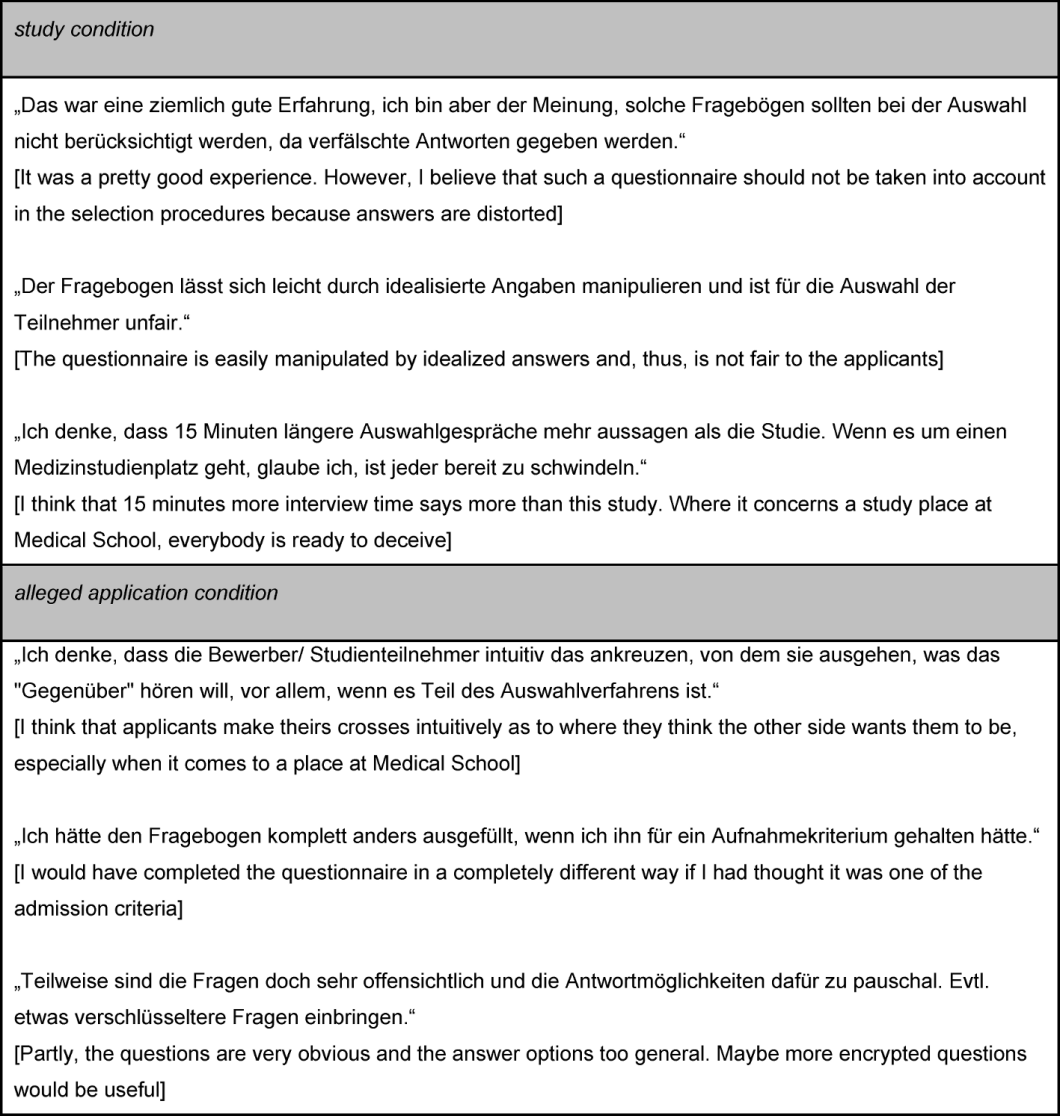
Comments from the evaluation of the interviews referring to the study
